# Factors associated with clinical nurses’ preconception health behavior in Korea: a cross-sectional survey

**DOI:** 10.4069/whn.2024.03.08

**Published:** 2024-03-29

**Authors:** Yoon-Jung Park, Sun-Hee Kim

**Affiliations:** 1 Nursing Division, Daegu Catholic University Medical Center, Daegu, Korea; 2 Research Institute of Nursing Science, Daegu Catholic University, College of Nursing, Daegu, Korea

**Keywords:** Health behavior, Health status, Nurses, Preconception care, Social support

## Abstract

**Purpose:**

Nurses have been reported to be at an increased risk for miscarriage and preterm labor. However, there is limited knowledge regarding nurses’ preconception health behaviors. Therefore, this study aimed to identify factors influencing these behaviors.

**Methods:**

One hundred sixty nurses, who were planning their first pregnancy within the upcoming year, participated in an online survey from August 11 to October 31, 2021. Data on preconception health behavior, perceived health status, pregnancy anxiety, nursing practice environment, and social support were analyzed using the t-test, Pearson correlation coefficients, and multiple regression analysis.

**Results:**

Age (*р*=.024), educational level (*р*=.010), marital status (*р*=.003), work experience (*р*=.003), satisfaction with the work department (*р*<.001), smoking status (*р*=.039), and previous health problems related to pregnancy outcomes (*р*=.004) were significantly associated with nurses’ preconception health behaviors. Furthermore, perceived health status (*р*<.001), pregnancy anxiety (*р*=.011), nursing practice environment (*р*=.003), and social support (*р*<.001) showed significant correlations with preconception health behaviors. Social support (β=.28, *р*=.001), satisfaction with the work department (β=.23, *р*=.032), marital status (β=.22, *р*=.002), and perceived health status (β=.23, *р*=.002) were confirmed as factors associated with preconception health behaviors. These factors explained 40.9% of the variance in preconception health behaviors (F=6.64, *р*<.001).

**Conclusion:**

Clinical nurses’ preconception health behaviors were influenced by social support, perceived health status, satisfaction with the work department, and marital status. Interventions to improve clinical nurses’ preconception health behaviors should target social support and perceived health status. A preconception health behavior education program considering clinical nurses’ marital status and satisfaction with the workplace can also be implemented.

## Introduction

Preconception care adopts a life-course approach, aiming to improve reproductive health even before conception [1]. For women to achieve a healthy pregnancy at their preferred timing, support is needed from their families, communities, and national health systems, encompassing intellectual, physical, and psychological preparation for pregnancy [2,3]. Women encounter various challenges prior to becoming pregnant, including delays and difficulties in conceiving, as well as associated physical and mental health issues [2]. Health problems related to pregnancy can have enduring effects on a woman’s overall well-being [4]. Therefore, the foundation for healthy pregnancy outcomes is laid through preconception care, since the period before pregnancy is a critical time for ensuring health [5].

Numerous studies have examined the factors associated with preconception health behaviors among women in the general population [6,7]. However, there is a paucity of studies focusing on the preconception health behaviors of nurses, a profession dominated by women. In Korea, approximately 72% of currently practicing nurses are in their 20s and 30s [8]. Considering that the average age of first marriage and childbirth in Korean women is 30.6 and 33.1 years, respectively [9], reproductive choices are likely to be relevant for the majority of nurses. Due to shift work and excessive workload, clinical nurses have lower levels of health behaviors than other professions and the general population [10], and they are at a higher risk of miscarriage and preterm labor [11]. Despite their role as professionals caring for the health of others, nurses frequently adopt a passive approach to their own health [10]. Therefore, identifying the factors that influence clinical nurses’ preconception health behaviors is crucial for promoting their well-being and ensuring the sustainability of their careers.

Factors related to health behavior can be categorized as personal and social [12]. Controllable personal factors include perceived health status [13] and pregnancy anxiety [14], while social factors encompass the nursing practice environment [6,15] and social support [6,7]. Perceived health status refers to individuals’ self-assessment of their overall health, which can contribute to health behaviors [16]. As women prepare for pregnancy, they become increasingly aware of their health issues and recognize the importance of preconception health behaviors [7]. Therefore, it is vital to ensure that clinical nurses accurately evaluate their own health status.

Furthermore, women of childbearing age often experience moderate to severe pregnancy anxiety, which is more prevalent among those who have never given birth [14]. Women frequently express anxiety about challenges in conceiving, pregnancy-related changes, miscarriage, and unknown fears [5]. Childbearing women also experience concerns about childbirth, appearance, body shape, and weight gain [17]. Health behaviors are also linked to anxiety experienced during pregnancy [18]. While some research has focused on pregnancy anxiety in childbearing women, there are limited studies exploring the relationship between this anxiety and health behaviors—particularly among reproductive-age clinical nurses.

Preconception health behaviors are influenced by social factors, such as the environment and available resources [6]. Specifically, the ability of clinical nurses to practice health behaviors is hindered by factors including shift work [19], constraints on time, the pressures of overwork, and the scarcity of resources and facilities [20]. Women working in healthcare are more likely to have miscarriages, preterm labor, and low-birth-weight deliveries than women in other professions [21]. Thus, it is necessary to investigate the relationship between preconception health behaviors and clinical nurses’ practice environment.

Social support from spouses, family, colleagues, and the community is instrumental in helping women achieve successful pregnancies and maintain psychological stability, which in turn encourages healthy behaviors before conception [6]. Pregnant nurses, in particular, draw strength from the understanding and support provided by head nurses and their peers, enabling them to continue working during their pregnancy [22]. Furthermore, higher levels of social support have been linked to improved health behaviors [20,23]. Collectively, previous research has shown that social support is an important factor for women preparing for pregnancy.

Previous studies on pregnant women have investigated the degree to which health behaviors are influenced by health status [24] and social support [25]. In addition, research on health behaviors among women of childbearing age has focused on factors including marriage and pregnancy awareness [26], fatigue, and depression [27]. However, there has been little research on preconception health behaviors among clinical nurses, a significant proportion of whom are reproductive-age women.

The purpose of this study was to investigate preconception health behavior, perceived health status, pregnancy anxiety, nursing practice environment, and social support among clinical nurses working in hospitals. Specifically, it aimed to clarify the relationships between these variables and identify the factors associated with preconception health behaviors.

## Methods

**Ethics statement:** This study was approved by the Institutional Review Board of Daegu Catholic University (CUIRB-2021-0029). Informed consent was obtained from the participants.

### Study design

This cross-sectional correlational study investigated clinical nurses’ preconception health behaviors, personal factors such as perceived health status and pregnancy anxiety, and social factors such as the nursing practice environment and social support. This study then explored the relationships among these factors to clarify their impact on preconception health behaviors. The study adhered to the STROBE (STrengthening the Reporting of OBservational studies in Epidemiology) guidelines (https://www.strobe-statement.org/).

### Participants

The participants in this study were clinical nurses employed in hospitals across Korea. The selection criteria included: women of childbearing age (between 20 and 49 years of age) who were currently working in a hospital with at least 12 months of clinical experience, in a relationship with a sexual partner, planning to conceive within the next year, and having no prior experience with pregnancy or childbirth. The sample size was calculated with the G*Power 3.1.9.7 program. Due to the lack of prior studies using a preconception health behavior measurement tool, this study assumed a significance level of .05, a power of .80, and a medium effect size of .15, with a total of 17 predictors, including 13 general characteristics and four main independent variables (perceived health status, pregnancy anxiety, nursing practice environment, and social support). The minimum sample size required for this study was determined to be 146 participants. Accounting for an anticipated 25% dropout rate due to the online survey format, the target sample size was set at 195. Of 220 responses, data from 33 ineligible participants (e.g., nurses working in screening clinics, counseling, or public health centers) and 27 surveys with mostly incomplete responses were excluded. As a result, the final sample size for analysis was 160 participants.

### Measurement

The study used instruments to assess preconception health behavior, perceived health status, pregnancy anxiety, nursing practice environment, and social support. These instruments were used with prior approval from the authors, obtained via e-mail.

#### Preconception health behavior

Preconception health behavior was assessed using a tool developed by Yeom and Kim [28], which was designed to evaluate the lifestyle and behavior of women preparing for pregnancy. It contains 27 items, including avoiding harmful substances (four items), professional healthcare (six items), rest and sleep (four items), stress management (four items), information acquisition (five items), and resource preparation (four items). Each item is scored on a 5-point Likert scale (1, not at all to 5, very much so). A higher score (possible range, 27 to 135 points) indicates a higher level of preconception health behavior. The tool’s reliability was shown by a Cronbach’s α of .92 when developed by Yeom and Kim [28], and it was .89 in this study.

#### Perceived health status

Perceived health status was based on the Health Perceptions Questionnaire developed by Ware [29] and modified and supplemented by Lee [30]. It consists of 20 items, including current health (seven items), past health (two items), future health (two items), health concerns (four items), resistance/susceptibility to illness (two items), and refusal of the patient role (three items). Each item is rated on a 4-point Likert scale (1, definitely false to 4 definitely true). A higher score (possible range, 20 to 80 points) indicates a better perceived health status. The reliability of the tool in Lee [30]’s study was Cronbach’s α=.72, while in this study, Cronbach’s α was .71.

#### Pregnancy anxiety

Pregnancy anxiety was measured using a tool developed by Huizink et al. [17] and translated by Kim [31]. It assesses anxiety about childbirth, having a child with disabilities, and physical changes. The tool includes 10 items: fear of childbirth (three items), fear of having a disabled child (four items), and concerns about one’s appearance (three items). Each item is scored on a 5-point Likert scale (1, not at all to 5, very much so). A higher score (from 10 to 50 points) indicates more pregnancy anxiety. The reliability was shown Cronbach’s α of .83 at development [17], while in this study, Cronbach’s α was .78.

#### Nursing practice environment

The Practice Environment Scale of the Nursing Work Index (PES-NWI), developed by Lake [32], measures nurses’ subjective feelings and attitudes toward the physical environment, peer interactions, and policies of the hospital. To assess the nursing practice environment in Korea, this study used the Korean version of the PES-NWI, which was modified by Cho et al. [33] for the Korean nursing context, and its reliability and validity were verified. This tool comprises 29 items, including four on adequate staffing and material support, nine on quality nursing foundations, nine on nurses’ participation in hospital operations, three on collaboration between nurses and doctors, and four on nursing managers’ capabilities, leadership, and support. Each item is scored on a 4-point Likert scale (1, not at all to 4, very much so), with a higher score (possible range, 29 to 116 points) indicating a more positive perception of the work environment. The reliability of the tool at the time of its development by Lake [32] was shown by a Cronbach’s α of .82, while in this study, Cronbach’s α was .91.

#### Social support

Social support was measured using a tool developed by Park [34], designed to assess subjective perceptions of various resources provided through relational bonds. This tool contains 25 items, including emotional support (seven items), informational support (six items), material support (six items), and appraisal support (six items). It utilizes a 5-point Likert scale (1, not at all to 5, very much so), with a higher score (possible range, 25 to 125 points) indicating a greater level of social support. Cronbach’s α in Park’s [34] study was .95, while it was .96 in this study.

#### General characteristics

The general characteristics of clinical nurses were measured with 13 items: age, educational level, religion, marital status, work schedule, work unit, work experience, average monthly salary, satisfaction with their work department, drinking status, smoking status, health problems, and health problems related to pregnancy outcomes.

### Study procedures

To mitigate potential discomfort and ensure candid responses, an online survey was used to explore clinical nurses’ personal experiences and perspectives on preconception health behaviors. The data were collected from August 11 to October 31, 2021. Participants were recruited via convenience sampling. The researchers explained the purpose and procedures of the study to the administrators of various nationwide nursing job and information exchange websites (e.g., Naver and Daum online communities, groups representing nurses and those preparing for the National Health Insurance Service exam) and obtained approval to post recruitment notices. Interested participants were directed to a URL linking to an online survey site (SurveyMonkey) created by the researchers. To prevent duplicate participation, the survey was set up to accept only one submission per IP address. The estimated time to complete the online survey was 20 minutes. As a token of appreciation, participants who completed the survey received a mobile coupon valued at about 3.5 US dollars, which was sent to their provided contact number.

### Data analysis

The collected data were analyzed using IBM SPSS for Windows ver. 28.0 (IBM Corp., Armonk, NY, USA), with statistical significance set at *p*>.05. The general characteristics of clinical nurses were examined in terms of frequency, percentage, mean, and standard deviation. Descriptive statistics were used to assess levels of perceived health status, pregnancy anxiety, nursing practice environment, social support, and preconception health behavior. The independent t-test, one-way analysis of variance, and the Scheffé test for post-hoc analysis were used to investigate differences in preconception health behavior based on clinical nurses’ general characteristics. The relationships between perceived health status, pregnancy anxiety, nursing practice environment, social support, and preconception health behavior were analyzed using Pearson correlations. Multiple regression analysis (simultaneous) was used to identify the factors that influenced clinical nurses’ preconception health behaviors.

## Results

### General characteristics of participants

Of the 160 participants, 80 nurses (50.0%) were between the ages of 30 and 34 years, and 116 (72.5%) had a bachelor’s degree. Ninety-six (60.0%) claimed no religious affiliation, and 136 (85.0%) were married. Shift workers accounted for 110 (68.8%) and 87 (54.4%) worked in general wards. The most common range of work experience was 5 to 9 years, reported by 69 nurses (43.1%). Ninety nurses (56.2%) had an average monthly salary of more than 3 million Korean won (approximately 2,200 US dollars), and 66 (41.3%) expressed moderate satisfaction with their work department. Seventy-eight nurses (48.8%) were former drinkers, and the majority (n=144, 90%) had never smoked. A total of 135 nurses (84.4%) reported no diagnosed health problems. For those who did report health problems, the conditions included thyroid disorders (n=10, 6.3%), heart diseases (n=3, 1.9%), depression (n=3, 1.9%), respiratory diseases (n=2, 1.3%), hypertension (n=1, 0.6%), and diabetes (n=1, 0.6%). Other conditions (n=10, 6.3%) accounted for hyperlipidemia, polycystic ovary syndrome, autoimmune diseases, and atopic dermatitis. Additionally, 122 nurses (76.3%) reported no health problems related to pregnancy outcomes (Table 1).

### Participants’ levels of perceived health status, pregnancy anxiety, nursing practice environment, social support, and preconception health behavior

The mean (±standard deviation) score for preconception health behavior was 100.68 (±12.62), indicating a moderate level. Perceived health status also showed a moderate mean score of 54.06 (±5.40), as did pregnancy anxiety, with a mean score of 37.26 (±5.94). The mean score for the nursing practice environment was 69.40 (±11.00), and social support averaged 89.75 (±15.89), both reflecting moderate levels (Table 2).

### Differences in preconception health behavior according to participants’ general characteristics

Significant differences in preconception health behavior were observed according to age (F=3.84, *p*=.024), educational level (F=4.70, *p*=.010), marital status (t=3.30, *p*=.003), work experience (F=5.99, *p*=.003), satisfaction with the work department (F=8.22, *p*<.001), smoking status (t=2.09, *p*=.039), and health problems related to pregnancy outcomes (t=2.90, *p*=.004) (Table 1).

### Relationships between preconception health behavior and participants’ perceived health status, pregnancy anxiety, nursing practice environment, and social support

This study found that participants’ preconception health behavior had significant weak to moderate positive correlations with perceived health status (r=.42, *p*<.001), pregnancy anxiety (r=.20, *p*=.011), nursing practice environment (r=.24, *p*=.003), and social support (r=.41, *p*<.001). Perceived health status showed weak positive correlations with pregnancy anxiety (r=.22, *p*=.006), nursing practice environment (r=.29, *p*<.001), and social support (r=.28, *p*<.001). Although there was no significant correlation between pregnancy anxiety and the nursing practice environment (r=.12, *p*=.135), it did have a weak positive correlation with social support (r=.27, *p*<.001). A weak positive correlation was found between the nursing practice environment and social support (r=.39, *p*<.001) (Table 3).

### Factors that influenced clinical nurses’ preconception health behavior

General characteristics that showed associations with participants’ preconception health behavior, including age, educational level, marital status, satisfaction with the work department, smoking status, and health problems related to pregnancy outcomes, were transformed into dummy variables. These were included in the regression analysis along with other variables that had significant correlations with preconception health behavior: perceived health status, pregnancy anxiety, nursing practice environment, and social support. The regression model was statistically significant (F=6.64, *p*<.001). The tolerance values ranged from 0.37 to 0.88, indicating no multicollinearity among the predictors, as the variance inflation factors ranged from 1.14 to 2.74, well below the threshold of 10. The Durbin-Watson statistic was close to 2 at 1.98, suggesting the absence of autocorrelation in the residuals. Furthermore, the regression standardized residual’s normal P-P plot demonstrated linearity, and the scatter plot revealed an even distribution of residuals around zero, confirming the normality and homoscedasticity of errors.

Social support (β=.28, *p*=.001), perceived health status (β=.23, *p*=.002), satisfaction with the work department (β=.23, *p*=.032), and marital status (β=.22, *p*=.002) significantly influenced preconception health behavior. Together, these factors accounted for 40.9% of the variation in clinical nurses’ preconception health behavior. Specifically, married individuals, those more satisfied with their department, and those with higher levels of perceived health status and social support were more likely to engage in preconception health behavior (Table 4).

## Discussion

This study found that the key factors influencing preconception health behaviors among clinical nurses included social support, perceived health status, satisfaction with their work department, and marital status. First and foremost, social support emerged as the most influential factor, consistent with previous research on its role in health behaviors [6]. This highlights the importance of social support in improving emotional and physical health behaviors among nurses. Preparing for pregnancy requires not only efforts made by the prospective mother and her partner, but also support from family, the workplace, and society [6]. Previous research has shown that social support can predict health behavior changes and maintenance [35]. Therefore, organizational strategies are needed to increase social support among nurses, who primarily consist of women of childbearing age [35]. Given that male partners may not actively engage in preconception health behaviors [5], it is crucial to raise their awareness about the importance of those behaviors and to expand educational opportunities for them.

Perceived health status was found to be the next major influencing factor. Higher levels of perceived health status were found to be associated with more positive preconception health behaviors. This finding is consistent with previous research indicating that higher perceived health status among nurses correlates with more favorable health behaviors [16]. Since preconception health issues can have a negative impact on birth outcomes, women planning to become pregnant should prioritize and maintain their health status [3]. In particular, clinical nurses, due to their work characteristics, are disproportionately likely to experience symptoms related to the digestive, nervous, and reproductive systems [36]. Therefore, educational programs should be developed with the goal of improving the perceived health status of clinical nurses preparing for pregnancy.

Third, participants who were more satisfied with their work department demonstrated better preconception health behaviors than those who were dissatisfied. This aligns with previous research finding that higher work department satisfaction was associated with increased general health promotion behaviors in operating room nurses [37]. However, direct comparisons to preconception health behaviors are difficult, highlighting the need for additional research. The nursing practice environment was not identified as an influencing factor in this study, despite previous research indicating a link between the nursing practice environment and health behaviors [37]. The discrepancy between the present study and previous research warrants further investigation.

Lastly, this study identified marital status as an influencing factor. Married nurses exhibited better preconception health behaviors than their unmarried counterparts, even those in a relationship with a sexual partner. This supports previous research, according to which married nurses engaged in more general health promotion behaviors [35]. A possible explanation for this finding is that marriage fosters mutual reliance and interest in each other’s health, resulting in improved health behaviors. Furthermore, because married nurses tend to be older and have more work experience, this study analyzed the relationships among marital status, age, and work experience. Nurses aged 35 years or older scored higher on preconception health behaviors than those aged 29 years or younger, and nurses with more than 5 years of work experience scored higher than those with 4 years or less of experience. The tendency in Korea for later marriages (e.g., after age 30 years) and a rise in pregnancies at an advanced maternal age suggest that the increased concern for health behaviors before conception may stem from the fear of infertility [38], along with the risks associated with miscarriage, preterm birth, and low-birth-weight deliveries [39]. Furthermore, more experienced nurses, often in leadership positions, might have inspired their colleagues by serving as role models for a healthy lifestyle [20]. Similar to the findings in this study, previous research [40] has shown that older nurses with more work experience exhibit better health behaviors. As a result, it is essential to emphasize the importance of preconception health behaviors and develop actionable strategies for nurses aged 29 years and younger and those with less than 5 years of work experience to effectively practice these behaviors.

Previous research suggests that individuals who frequently experience anxiety symptoms are more likely to engage in less physical activity, suffer from inadequate sleep, and adopt unhealthy behaviors such as excessive drinking and smoking [41]. However, pregnancy anxiety was not found to be a significant predictor of preconception health behaviors in this study. A reason for this discrepancy may be that the participants in this study did not explicitly report anxiety symptoms, and it is also possible that pregnancy anxiety did not directly impact preconception health behaviors because the participants were not yet pregnant. Consequently, women of childbearing age may find it difficult to adopt preconception health behaviors due to uncertainties regarding when they will conceive. Nonetheless, the motivation to change these behaviors generally increases after pregnancy is confirmed.

In this study, clinical nurses had a mean score of 100.68 points (item mean, 3.73) on preconception health behaviors, which is lower than the 114.17 points (item mean, 4.23) reported in a previous study on women of childbearing age [42]. Other research has also shown that nurses exhibit lower levels of health behaviors than those in other professions [43]. Nurses are aware of the need for their own health behaviors; however, it is difficult for them to maintain regular health behaviors due to factors including a lack of time, excessive workload, limited facilities for physical activities, insufficient cooking appliances for accessing healthy food options, fatigue, and lack of sleep [20]. Furthermore, approximately 70% of the participants in this study worked as shift nurses, which may have influenced their preconception health behaviors. Existing research suggests a link between shift work and poorer health behaviors. For instance, shift-working nurses were found to consume more fats and saturated fats than day-shift nurses [44], and among smoking nurses, those working shifts were more likely to continue smoking during pregnancy than their day-shift counterparts [45]. This highlights the need for further research on the factors contributing to the lower levels of preconception health behaviors among shift-working nurses.

This study is significant because, to the best of our knowledge, it is the first to investigate the factors influencing preconception health behaviors among clinical nurses, who are predominantly women. Furthermore, it distinguishes between personal and social factors, such as perceived health status and social support, respectively. Based on these findings, we propose developing educational programs and interventions aimed at strengthening nurses’ perceived health status and social support to improve preconception health behaviors among clinical nurses, considering marital status and satisfaction with the work unit. Although the factors identified in this study explained only a moderate portion (40.9%) of the variance in preconception health behaviors, previous research has also found other influencing factors, such as motivation and the financial costs of health behaviors [7], which warrant further investigation in future studies. Furthermore, because the data were collected among clinical nurses through convenience sampling from selected online platforms, there is a potential bias favoring users of these platforms. This limitation may restrict the generalizability of these findings. Therefore, future research should broaden the study population to include more diverse groups of women of childbearing age, shift-working women, and their partners.

## Figures and Tables

**Table 1.** General characteristics of clinical nurses and differences in preconception health behavior (N=160)

Variable	Categories	n (%)	Mean±SD	t/F	*р*	Scheffé
Age (year)	≤29^a^	53 (33.1)	97.49±12.13	3.84	.024	a<c
	30-34^b^	80 (50.0)	101.18±13.10			
	≥35^c^	27 (16.9)	105.48±10.64			
Education level	Diploma^a^	29 (18.1)	96.21±12.71	4.70	.010	a<c
	Bachelor’s^b^	116 (72.5)	100.83±11.71			
	≥Master’s^c^	15 (9.4)	108.20±16.04			
Religious affiliation	Yes	64 (40.0)	101.47±13.62	0.64	.521	
	No	96 (60.0)	100.16±11.96			
Marital status	Unmarried	24 (15.0)	91.33±15.61	3.30	.003	
	Married	136 (85.0)	102.33±11.31			
Work schedule	Shift work	110 (68.8)	99.89±12.23	1.18	.241	
	Fixed work	50 (31.2)	102.42±13.42			
Work unit	General ward	87 (54.4)	99.02±12.48	1.75	.178	
	Special ward^†^	55 (34.4)	102.31±11.92			
	Outpatient	18 (11.2)	103.72±14.76			
Work experience (year)	≤4^a^	44 (27.5)	95.41±13.96	5.99	.003	a<b, c
	5-9^b^	69 (43.1)	101.88±11.34			
	≥10^c^	47 (29.4)	103.85±11.82			
Average monthly salary (Korean won)^‡^	<3 million	70 (43.8)	98.89±12.48	1.59	.113	
	≥3 million	90 (56.2)	102.08±12.63			
Satisfaction with the work department	Dissatisfied^a^	33 (20.6)	94.85±12.94	8.22	<.001	a<c
	Neutral^b^	66 (41.3)	99.52±12.53			
	Satisfied^c^	61 (38.1)	105.10±11.09			
Drinking status	Current drinker	67 (41.9)	99.46±13.86	0.56	.571	
	Former drinker	78 (48.8)	101.42±10.58			
	Never-drinker	15 (9.3)	102.27±16.60			
Smoking status	Current or former smoker	16 (10.0)	94.50±14.94	2.09	.039	
	Never-smoker	144 (90.0)	101.37±12.21			
Disease	Yes	25 (15.6)	99.88±11.96)	–0.35	.731	
	No	135 (84.4)	100.83±12.78			
Previous health problems related to pregnancy outcomes	Yes	38 (23.7)	105.67±12.95	2.90	.004	
	No	122 (76.3)	99.07±12.14			

† Emergency room, intensive care unit, operating room, delivery room, and newborn nursery.

‡ 3 million Korean won is roughly 2,250 US dollars.

**Table 2.** Degrees of preconception health behaviors, perceived health status, pregnancy anxiety, nursing practice environment, and social support in clinical nurses (N=160)

Variable	Number of items	Possible range	Mean±SD
Preconception health behavior	27	27–135	100.68±12.62
Perceived health status	20	20–80	54.06±5.40
Pregnancy anxiety	10	10–50	37.26±5.94
Nursing practice environment	29	29–116	69.40±11.00
Social support	25	25–125	89.75±15.89

**Table 3.** Correlations among preconception health behavior, perceived health status, pregnancy anxiety, nursing practice environment, and social support (N=160)

Variable	r (*р*)			
	Preconception health behavior	Perceived health status	Pregnancy anxiety	Nursing practice environment
Preconception health behavior	1			
Perceived health status	.42 (<.001)	1		
Pregnancy anxiety	.20 (.011)	.22 (.006)	1	
Nursing practice environment	.24 (.003)	.29 (<.001)	.12 (.135)	1
Social support	.41 (<.001)	.28 (<.001)	.27 (<.001)	.39 (<.001)

**Table 4.** Factors associated with clinical nurses’ preconception health behavior (N=160)

Variable	B	SE	β	t	*p*
(Constant)	41.94	9.93		4.23	.000
Age 1 (30–34 years)^†^	–0.05	2.18	0.00	–0.02	.981
Age 2 (≥35 years)^†^	2.19	3.19	0.07	0.69	.493
Education level 1 (bachelor’s)^†^	1.86	2.20	0.07	0.85	.399
Education level 2 (≥master’s)^†^	3.36	3.52	0.08	0.96	.341
Marital status (married)^†^	7.80	2.46	0.22	3.17	.002
Work experience 1 (5–9)^†^	3.65	2.20	0.14	1.66	.099
Work experience 2 (≥10)^†^	4.06	2.93	0.15	1.39	.168
Satisfaction with the work department 1 (neutral)^†^	2.23	2.41	0.09	0.93	.356
Satisfaction with the work department 2 (satisfied)^†^	5.88	2.72	0.23	2.16	.032
Smoking status (current or former smoker)^†^	0.14	2.87	0.00	0.05	.961
Previous health problems related to pregnancy outcomes (yes)^†^	3.78	2.00	0.13	1.89	.061
Perceived health status	0.55	0.17	0.23	3.12	.002
Pregnancy anxiety	0.02	0.15	0.01	0.13	.894
Nursing practice environment	–0.10	0.10	–0.09	–1.02	.308
Social support	0.22	0.06	0.28	3.43	.001
R²=.41, adjusted R²=.35, F=6.64, *р*<.001					

B, Unstandardized regression coefficient; SE, standard error; β, standardized regression coefficient.

† The reference values were age 1 (≤29 and ≥35 years), age 2 (≤34 years), education level 1 (diploma and master’s or higher), education level 2 (diploma and bachelor’s), marital status (unmarried), work experience 1 (≤4 and ≥10 years); work experience 2 (≤9 years), satisfaction with the work department 1 (dissatisfied and satisfied), satisfaction with the work department 2 (dissatisfied and neutral), smoking status (never-smoker), and previous health problems related to pregnancy outcomes (none).
